# Maintaining the working state of firefighters by utilizing self-concept clarity as a resource

**DOI:** 10.1186/s12889-024-17896-1

**Published:** 2024-02-02

**Authors:** Peng Wu, Tingting Liu, Qingqian Li, Xiaoting Yu, Zhiyun Liu, Siyu Tian

**Affiliations:** 1 Tianjin University of Sport, Tianjin, China; 2 Tianjin Medical University, Tianjin, China; 3 Jinan Engineering Polytechnic, Jinan, China; 4 School of Management, Shandong University, Jinan, China; 5 School of Physical Education, Shandong University, Jinan, China

**Keywords:** Self-concept clarity, Job burnout, Work engagement, Resilience, Job demand–resource theory

## Abstract

The working state of firefighters is important for their own safety as well as that of the general public. The purpose of this study is to investigate the correlations between self-concept clarity, resilience, work engagement, and job burnout among firefighters, as well as the impacts of self-concept clarity and resilience as resources that can maintain their working state. Based on data from 2,156 firefighters, analysis showed that self-concept clarity was negatively associated with job burnout and positively associated with work engagement. The results also showed that self-concept clarity had a direct effect on job burnout and work engagement, and an indirect effect by improving the firefighters’ resilience. Maintaining and improving their self-concept clarity and resilience promises to be an effective strategy for guaranteeing the working state of firefighters.

## Introduction

People in the workplace are generally facing increasing pressure and fatigue at the same time, while also striving to maintain a high level of enthusiasm and vitality. These two psychological and behavioral states in work are referred to as job burnout and work engagement, respectively [1, 2]. Job burnout is a prolonged negative response which is caused by the gradual physical and mental exhaustion of individuals who are unable to manage effectively long-term pressure at work [3]. In general, job burnout is considered to be composed of three dimensions: exhaustion, cynicism, and reduced professional efficacy [4]. In contrast, work engagement refers to an individual’s active integration into work from physiological, cognitive, and emotional perspectives [5].

Firefighters have to face adverse, unpredictable, and demanding situations every day, and suffer great pressure at work, which makes it difficult for them to maintain a good working condition [6]. Research has shown that firefighters who experience job burnout are more likely to ignore safety regulations and violate standard operating practices [7]. Thus, the consequences of burnout can harm firefighters when they are performing their duties [8]. In contrast, work engagement is very important for improving work ability [9, 10]. When firemen are engaged at work, they tend to perform better on the job [11, 12]. Therefore, an essential research question is how to reduce job burnout among firefighters while also improving their work input and working state.

Due to the importance of maintaining the working state of firefighters, a large number of studies have explored the influential factors. Previous studies have shown that many health-related quality of life factors, both psychological and physical, affect job burnout in firefighters, and that gender and education levels may also contribute to differences in the degree of burnout among firefighters [13]. In addition, work pressure, work-family conflict, achievement goals and coping strategies are factors that also predict job burnout among firefighters [14–16]. On the other hand, factors that influence firefighters’ work engagement include organizational-level demands and work meaning [11, 17].

Job Demand–Resource (JD–R) theory is a classical theory used to understand and explain job burnout and work engagement [1, 18]. From this theoretical perspective, research has included proactive coping and support among the factors that influence the working state of firefighters [17, 19, 20]. However, there is no research on firefighters that includes self-clarity under the framework of JD–R theory, which is a factor worthy of research and potential application.

## Theoretical framework and hypotheses

### Job demand–resource theory

JD–R theory suggests that a lack of job resources will lead to job burnout; conversely, job resources available to individuals will promote work engagement [2, 21, 22]. Moreover, the dual-process model also points out that job demands can only lead to job burnout, while abundant job resources and personal resources can not only slow down the burnout process and reduce job burnout, but also enhance work engagement, especially in the context of high job requirements [23, 24]. Therefore, in order to protect the working state of firefighters, more attention should be paid to the development of their individual resources. Self-concept clarity and resilience are both important individual resources, and can be considered to play a role in maintaining and improving the working state of firefighters.

### The relationship between self-concept clarity and working state

Self-concept clarity as proposed by Campbell et al. [25] is an important personal resource, which refers to the extent to which an individual’s self-concept is clearly defined, and also to the internal consistency and temporal stability of their self-concept definition. Self-concept clarity reflects the individual’s structural integrity of self-concept, and is a kind of metacognitive awareness of “the structural integrity of self-belief” [25, 26]. Previous studies have shown that higher levels of self-concept clarity have an effect on reducing burnout in athletes [27]. In addition, it is also an important factor in promoting the development of student and athlete engagement [27, 28]. Based on studies in student and athlete populations, it can be considered that self-concept clarity plays an active role in mitigating burnout and improving engagement. However, the relationships between self-concept clarity and job burnout and work engagement have not been tested directly. There is also a lack of studies examining the relationship between self-concept clarity and the three dimensions of job burnout (exhaustion, cynicism, and reduced professional efficacy). Therefore, it is essential to explore the link between self-concept clarity and firefighters’ job burnout and work engagement in order to verify its benefits as a personal resource for their working state. Based on the above, the following research hypotheses are proposed:

- *Hypothesis 1*: Self-concept clarity is negatively correlated with job burnout (exhaustion, cynicism, and reduced professional efficacy) among firefighters.
- *Hypothesis 2*: Self-concept clarity is positively correlated with work engagement among firefighters.

### The mediating role of resilience in the relationship between self-concept clarity and working state

Resilience is often manifested as a process by which individuals are able to adjust effectively in the face of adversity, trauma, or severe stress [29–31], which is similar to the outcome of promoting the development of individual adaptive functions brought by higher self-concept clarity [25, 32]. Liang et al. [33] demonstrated that self-concept clarity can positively affect individual resilience, while Heydari et al. [34] pointed out that resilience is extremely important for firefighters, and it is therefore necessary to explore its antecedent factors. Previous studies have found that support and self-encouragement are effective predictors of firefighters’ resilience [35, 36]. However, the association between self-concept clarity and resilience in firefighters has not been demonstrated, and the cited research needs to be supplemented.

Additionally, in research that has expanded JD–R theory, resilience has been regarded as an important resource that that can improve an individual’s working state [37], being both a protective factor against job burnout and a positive predictor of work engagement [38, 39]. It has been pointed out that people with higher self-concept clarity have greater psychological regulation and are better able to use internal resources to quickly adapt to challenging situations as well as to deal with emotions [25, 32, 40]. Hence, self-concept clarity might not only be a straightforward personal resource but may also contribute to the cultivation of other job resources, such as resilience, and thus safeguard the individual’s working state indirectly. Furthermore, as an individual resource, self-concept clarity may also affect an individual’s working state in different ways besides the pathway of resilience. Therefore, resilience may be more likely to partially mediate the relationship between self-concept clarity and working state. Based on the above considerations, the following hypotheses are proposed:

- *Hypothesis 3*: Self-concept clarity is positively correlated to resilience in firefighters.
- *Hypothesis 4*: Resilience mediates the relationship between firefighters’ self-concept clarity and working state (job burnout, work engagement).

### The present study

In summary, the job of a firefighter is stressful and demanding. In order to protect their physical and mental health, and to ensure good work performance, maintaining a good working state among firefighters is a high priority. The aim of the present study is to examine the potential of self-concept clarity as a resource and to extend the Job Demand–Resource model. In addition, on the basis of verifying the relationship between self-concept clarity, resilience, and working state (job burnout, work engagement), this paper attempts to analyze further the mechanism of self-concept clarity, and to provide valuable theoretical and practical guidance by exploring the mediating role of resilience.

## Materials and methods

### Participants and procedure

The subjects of this study were male firefighters. Twenty-two fire brigades and 130 fire stations in Tianjin, China, were chosen as sample units. Utilizing cluster sampling, the research obtained data by contacting each fire brigade commander. The Ethics Committee of Shandong University of Nursing and Rehabilitation approved the project (reference id: 2022-R-99). Informed consent was obtained from all subjects and/or their legal guardian(s). All respondents were also given the option to accept or reject participation in the study before the poll and were told of its objective. A total of 2227 questionnaires were distributed, and 71 were disqualified due to missing values. The efficacy percentage of the surveys was 96.81%.

### Measures

#### Self-Concept Clarity Scale (SCCS)

To assess participants’ self-concept clarity, we utilized the Chinese version of the Self-Concept Clarity Scale (SCCS) developed by Campbell et al. [25] and translated by Niu et al. [41]. The 12-item scale employs Likert’s five-point scoring system, with 1 denoting “strongly disagree” and 5 denoting “strongly agree”. Item 6 and Item 11 are unexceptionally scored, while the remaining items are reverse-rated. The respondent’s self-concept clarity increases with higher scores. The Cronbach’s alpha coefficient for the scale was 0.870. The scale also had good construct validity (KMO = 0.944, *p* < 0.001).

#### The 14-Item resilience scale (RS-14)

To assess the resilience of participants, the Chinese version of the 14-Item Resilience Scale (RS-14) was used [42]. The scale consists of 14 items and adopts a seven-point Likert-style rating system, with 1 indicating “very inconsistent” and 7 indicating “very consistent”. The higher the score, the stronger the resilience of the subjects. In this investigation, Cronbach’s alpha coefficient of the scale was 0.982, and the KMO value was 0.973 (*p* < 0.001), which indicated that the scale had good reliability and validity.

#### Maslach burnout inventory general survey (MBI-GS)

To assess job burnout among the participants, a Chinese version of the Maslach Burnout Inventory General Survey (MBI-GS) was used [43]. The measure uses a 5-point scale, with 0 representing “never” and 4 representing “daily”. The inventory consists of 16 items, including the 3 dimensions of exhaustion (5 items), cynicism (5 items), and professional efficacy (6 items) [44]. The items of the professional efficacy subscale are scored in reverse. A higher score in each dimension indicates a higher level of exhaustion, cynicism, and reduce professional efficacy, respectively. In this study, Cronbach’s alpha coefficient for the exhaustion subscale was 0.937, and the KMO value was 0.894 (*p* < 0.001). For the cynicism subscale, Cronbach’s alpha was 0.800 and the KMO value was 0.772 (*p* < 0.001). For the professional efficacy subscale, Cronbach’s alpha was 0.946 and the KMO value was 0.901 (*p* < 0.001).

#### Utrecht work engagement scale short version (UWES-9)

To assess participants’ work engagement, we used a Chinese translation of the short version of the Utrecht Work Engagement Scale (UWES-9), which was created by Schaufeli et al. [45]. The scale contains 9 items and uses a seven-point rating system, with 1 representing “never” and 7 representing “always”. The higher the score, the higher the participant’s level of work engagement. Cronbach’s alpha coefficient in this study was 0.982. The scale had good construct validity (KMO = 0.958, *p* < 0.001).

### Statistical analyses

SPSS 24.0 was used to conduct all statistical analyses (IBM; Armonk, NY, USA). Common method bias was calculated using the Harman single factor test. Pearson correlation coefficients were used to evaluate the correlations between the variables and age, years in work, and number of police callouts per week, respectively. Independent *t*-tests were used to compare the differences in the variables between the firefighters’ location, injury history, and children vs. no children. ANOVA tests were used to compare the differences in the variables between marital status and education level, and the results were further analyzed by multiple comparison analysis. Demographic variables that were significantly associated with the study variables were included as covariates in subsequent regression analysis models. The means and standard deviations of the variables were calculated using descriptive statistics, and Pearson correlation analysis was utilized to determine the associations between them. The mediation model was assessed by regression analysis using the Hayes PROCESS macro (version 3.0), with demographic data that were significantly associated with the study variables as covariates. Each variable was standardized before being entered into the mediation model. The effects were computed using PROCESS based on 5000 bootstrap samples, and a 95% deviation-corrected confidence interval (CI) was generated. Since the CI did not contain zero, the impact was seen to be meaningful. The level of significance was set at 0.05.

## Results

### Common method bias testing

The results of the unrotated factor solutions tests using exploratory component analysis discovered six factors with eigenvalues larger than one. The covariance ratio of the first factor between each measure was 37.166%, which is below the threshold criterion of 40% [46]. The findings demonstrate that the prevalent methodological bias in the current study did not cause any major issues.

### Demographic statistics and differences

There were 2,156 male firefighters in the final sample. Their average age was 26.94 ± 4.47 years and their average years in work was 5.92 ± 4.37 years. The average number of police callouts per week was 5.68 ± 7.01. Among the participants, 1,331 (61.73%) were unmarried, 811 (37.62%) were married, and 14 (0.65%) were divorced. Those with children numbered 564 (26.16%), while 1,592 (73.84%), were without. As regards location, 904 (41.93%) lived in urban areas and 1,252 (58.07%) lived in rural areas. There were 459 with a secondary education (21.29%), 1,285 with a junior college education (59.60%), 392 with a bachelor’s degree (18.18%), and 20 with a master’s degree or above (0.93%). Finally, 390 (18.09%) had an injury experience, while 1766 (81.91%) had no injury experience.

Table 1 shows the association of demographic variables with firefighters’ self-concept clarity, resilience, job burnout (exhaustion, cynicism, and reduced professional efficacy), and work engagement. The results show a significant correlation between age and resilience (*p* < 0.01), exhaustion (*p* < 0.05), and cynicism (*p* < 0.05), respectively. Older firefighters tend to have lower resilience, higher exhaustion, and higher cynicism. Years in work were also significantly associated with resilience (*p* < 0.05), exhaustion (*p* < 0.01), and cynicism (*p* < 0.05), respectively. The longer the period in work, the lower the resilience, and the higher the exhaustion and cynicism. There were group differences in exhaustion (*F* = 3.95, *p* < 0.05) and cynicism (*F* = 3.48, *p* < 0.05) among firefighters with different marital status. Analysis of multiple comparisons after ANOVA showed that the levels of exhaustion (*p* < 0.01) and cynicism (*p* < 0.01) among married firefighters were significantly higher than those among unmarried firefighters.

**Table 1:** The associations of demographic variables with self-concept clarity, resilience, job burnout (exhaustion, cynicism, and reduced professional efficacy), and work engagement in firefighters

Variables	Age	Work years	Police calls	Marital status			Presence or absence of children		Location		Education level				Injury history	
								**Absent**	**Urban**	**Rural**	**Secondary education**	**Junior college education**	**Bachelor's degree**	**Master’s degree or above**	**Yes**	**No**
				**Mean (*SD*)**	**Mean (*SD*)**	**Mean (*SD*)**	**Mean (*SD*)**	**Mean (*SD*)**	**Mean (*SD*)**	**Mean (*SD*)**	**Mean (*SD*)**	**Mean (*SD*)**	**Mean (*SD*)**	**Mean (*SD*)**	**Mean (*SD*)**	**Mean (*SD*)**
Self-concept clarity	0.01	0.04	-0.02	40.81	40.75	41.71	40.43	40.93	41.25	40.46	39.88*	40.74*	41.92*	43.20*	39.14***	41.16***
				(9.90)	(10.11)	(9.96)	(10.21)	(9.88)	(9.94)	(9.98)	(10.12)	(10.03)	(9.70)	(4.69)	(9.14)	(10.11)
Resilience	-0.06**	-0.05*	-0.01	74.91	72.88	70.36	73.05	74.50	74.19	74.07	72.11	74.57	75.05	72.85	70.53***	74.91***
				(21.31)	(21.59)	(22.12)	(21.70)	(21.34)	(21.39)	(21.48)	(22.86)	(21.42)	(19.68)	(20.64)	(20.24)	(21.62)
Exhaustion	0.05*	0.07**	0.04	4.58*	5.24*	4.50*	5.02	4.76	5.07	4.66	4.29***	4.71***	5.77***	6.80***	7.22***	4.20***
				(5.13)	(5.47)	(5.02)	(5.35)	(5.23)	(5.33)	(5.21)	(5.22)	(5.17)	(5.48)	(5.52)	(5.70)	(5.01)
Cynicism	0.05*	0.05*	0.04	3.84*	4.36*	4.21*	4.26	3.96	4.03	4.05	3.70***	3.89***	4.78***	6.70***	5.78***	3.65***
				(4.36)	(4.45)	(4.32)	(4.40)	(4.40)	(4.28)	(4.49)	(4.35)	(4.34)	(4.54)	(4.60)	(5.12)	(4.13)
Reduced professional efficacy	0.01	-0.01	0.01	7.73	7.85	7.57	8.05	8.28	7.65	7.87	8.67*	7.62*	7.22*	8.05*	8.30	7.66
				(8.16)	(8.06)	(8.16)	(7.68)	(8.07)	(7.93)	(8.26)	(8.84)	(8.13)	(7.24)	(4.76)	(7.34)	(8.28)
Work engagement	-0.03	-0.04	-0.03	47.87	46.75	46.43	46.74	47.68	47.15	47.64	47.12	47.86	46.48	36.80	43.97***	48.20***
				(15.81)	(15.58)	(17.01)	(15.59)	(15.78)	(15.39)	(15.98)	(16.93)	(15.55)	(14.93)	(14.05)	(14.95)	(15.80)

Pearson correlation coefficients were used to evaluate the correlations of the variables with age, years in work, and number of police callouts per week, respectively. Independent *t*-tests were used to compare the differences in variables between presence vs. absence of children, firefighter location, and injury history respectively. ANOVA was used to compare the differences in variables between marital status and education level, respectively. * *p* < 0.05; *** *p* < 0.001

As for educational level, significant differences were shown in self-concept clarity (*F* = 3.35, *p* < 0.05), exhaustion (*F* = 7.02, *p* < 0.001), cynicism (*F* = 7.55, *p* < 0.001), and reduced professional efficacy (*F* = 2.62, *p* < 0.05). The results of multiple comparisons analysis showed that firefighters with a bachelor’s degree (*p* < 0.05) and those with a master’s degree or above (*p* < 0.05) both had significantly higher levels of self-concept clarity than those with secondary education. At the same time however, those with a bachelor’s degree (*p* < 0.001) and those with a master’s degree or above (*p* < 0.05) both had significantly higher levels of exhaustion than those with secondary education, while those with a bachelor’s degree had a significantly higher level of exhaustion than those with junior college education (*p* < 0.001). In terms of cynicism, those with a bachelor’s degree (*p* < 0.001) and those with a master’s degree or above (*p* < 0.01) scored significantly higher than those with secondary education, while those with a bachelor’s degree (*p* < 0.001) and those with a master’s degree or above (*p* < 0.01) scored significantly higher than those with junior college education. As for reduced professional efficacy, only those with a bachelor’s degree scored significantly lower than those with junior college education (*p* < 0.05), and there were no differences between other education levels. In addition, compared with injured firefighters, uninjured firefighters had higher levels of self-concept clarity (*t* =  − 3.88, *p* < 0.001), resilience (*t* =  − 3.82, *p* < 0.001), and work engagement (*t* =  − 4.84, *p* < 0.001), and lower levels of exhaustion (*t* = 9.32, *p* < 0.001) and cynicism (*t* = 7.66, *p* < 0.001).

### Correlation analysis of self-concept clarity, resilience, and working state

Table 2 shows the mean values, standard deviations, and correlation matrices of self-concept clarity, resilience, job burnout (exhaustion, cynicism, and reduced professional efficacy), and work engagement. Self-concept clarity was negatively correlated with firefighters’ exhaustion, cynicism, and reduced professional efficacy, but was positively correlated with firefighters’ resilience and work engagement. Resilience had a positive relationship with work engagement and a negative relationship with exhaustion, cynicism, and reduced professional efficacy. Exhaustion, cynicism, and reduced professional efficacy were negatively correlated with work engagement. These results support Hypotheses 1, 2, and 3. Significant correlations between variables (all *p* < 0.01) provided a solid basis for subsequent regression analysis and mediation analysis.

**Table 2:** Descriptive statistics and correlation matrix for self-concept clarity, resilience, job burnout (exhaustion, cynicism, and reduced professional efficacy), and work engagement

	1	2	3	4	5	6
1. Self-concept clarity	1.00					
2. Resilience	0.22**	1.00				
3. Exhaustion	− 0.37**	− 0.23**	1.00			
4. Cynicism	− 0.35**	− 0.19**	0.75**	1.00		
5. Reduced professional efficacy	− 0.18**	− 0.48**	− 0.07**	− 0.10**	1.00	
6. Work engagement	0.24**	0.62**	− 0.27**	− 0.25**	− 0.51**	1.00
*M*	40.79	74.12	4.83	4.04	7.78	47.44
*SD*	9.97	21.44	5.26	4.4	8.12	15.73

^**^ *p* < 0.01

### Regression analysis

**Fig. 1:**
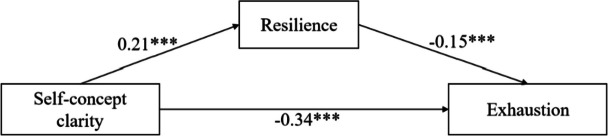
Resilience as a mediator between self-concept clarity and exhaustion. The values are the *β* coefficients of the path, with age, years in work, injury history, education level, and marital status as covariances. *** *p* < 0.001

**Fig. 2:**
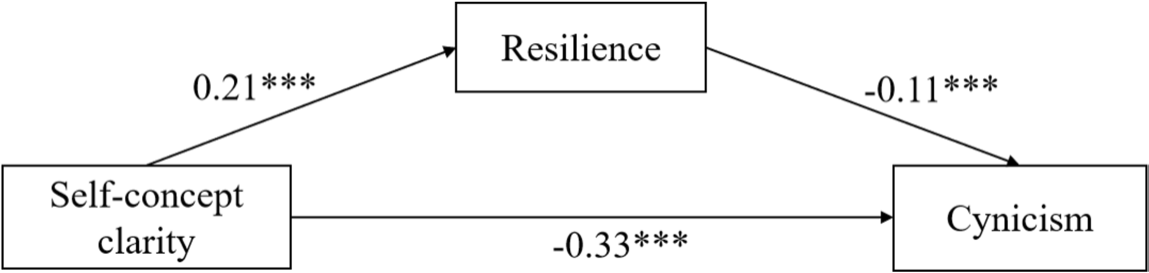
Resilience as a mediator between self-concept clarity and cynicism. The values are the *β* coefficients of the path, with age, years in work, injury history, education level, and marital status as covariances. *** *p* < 0.001

**Fig. 3:**
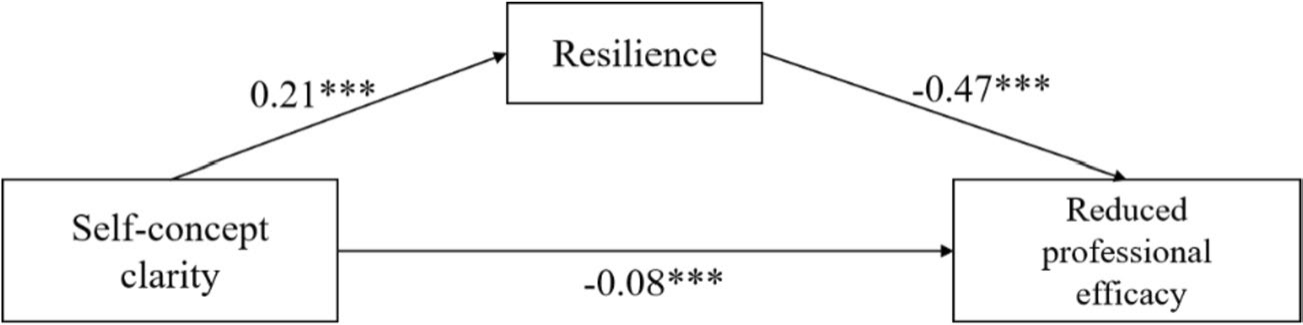
Resilience as a mediator between self-concept clarity and reduced professional efficacy. The values are the *β* coefficients of the path, with age, years in work, injury history, education level, and marital status as covariances. *** *p* < 0.001

The regression coefficients between self-concept clarity, resilience, and job burnout (exhaustion, cynicism, and reduced professional efficacy) are displayed in Table 3. In the exhaustion model, self-concept clarity negatively predicted exhaustion (*β* =  − 0.34, *p* < 0.001), and positively predicted resilience (*β* = 0.21, *p* < 0.001). Additionally, resilience negatively predicted exhaustion (*β* =  − 0.15, *p* < 0.001). In the cynicism model, self-concept clarity had a significant negative effect on cynicism (*β* =  − 0.33, *p* < 0.001) and a significant positive effect on resilience (*β* = 0.21, *p* < 0.001). Additionally, resilience has a significant negative effect on cynicism (*β* =  − 0.11, *p* < 0.001). The results of the reduced professional efficacy model showed that the path coefficient between self-concept clarity and resilience (*β* = 0.21, *p* < 0.001) was positively significant. The path coefficient between self-concept clarity and reduced professional efficacy (*β* =  − 0.08, *p* < 0.001) was negatively significant, as was the path coefficient between resilience and reduced professional efficacy (*β* =  − 0.47, *p* < 0.001). These results show that resilience partially mediates the relationship between self-concept clarity and job burnout (Figs. 1, 2 and 3).

**Table 3:** Regression coefficients of the mediation of resilience between self-concept clarity and job burnout

Model	Outcome variables	Predictive variables	Goodness-of-fit indices			Regression coefficient and significance	
			*R*	*R*^*2*^	*F*	*β*	*t*
Exhaustion	Resilience		0.24	0.06	22.19***		
		Self-concept clarity				0.21	10.12***
	Exhaustion		0.46	0.21	80.16***		
		Self-concept clarity				− 0.34	− 17.02***
		Resilience				− 0.15	− 7.53***
Cynicism	Resilience		0.24	0.06	22.19***		
		Self-concept clarity				0.21	10.12***
	Cynicism		0.42	0.17	63.89***		
		Self-concept clarity				− 0.33	− 16.12***
		Resilience				− 0.11	− 5.45***
Reduced professional efficacy	Resilience		0.24	0.06	26.63***		
		Self-concept clarity				0.21	10.12***
	Reduced professional efficacy		0.49	0.24	114.17***		
		Self-concept clarity				− 0.08	− 3.97***
		Resilience				− 0.47	− 24.18***

^***^ *p* < 0.001

**Fig. 4:**
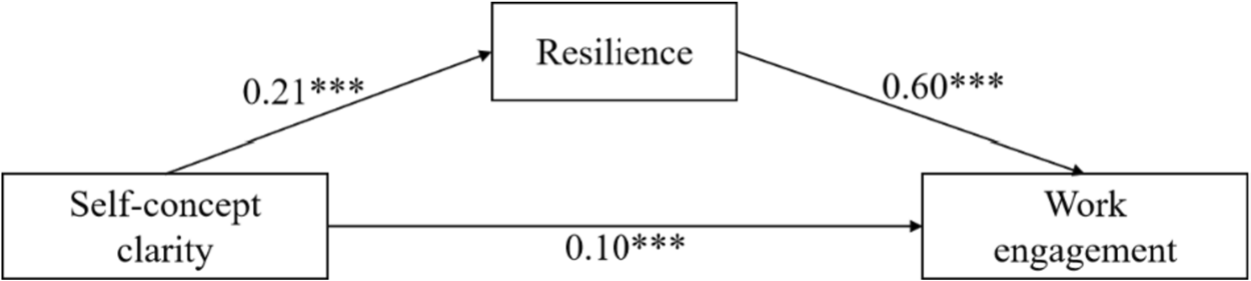
Resilience as a mediator between self-concept clarity and work engagement. The values are the *β* coefficients of the path, with age, years in work, injury history, education level, and marital status as covariances. *** *p* < 0.001

Table 4 shows the regression coefficients between self-concept clarity, resilience, and work engagement. The results show that self-concept clarity positively predicted work engagement (*β* = 0.10, *p* < 0.001) and resilience (*β* = 0.21, *p* < 0.001). Also, resilience was a significant positive predictor of work engagement (*β* = 0.60, *p* < 0.001). The findings indicate that resilience partially mediated the relationship between self-concept clarity and work engagement (Fig. 4).

**Table 4:** Regression coefficients of the mediation of resilience between self-concept clarity and work engagement

Outcome variables	Predictive variables	Goodness-of-fit indices			Regression coefficient and significance	
		*R*	*R*^*2*^	*F*	*β*	*t*
Resilience		0.24	0.06	25.63***		
	Self-concept clarity				0.21	10.12***
Work engagement		0.63	0.40	239.16***		
	Self-concept clarity				0.10	6.08***
	Resilience				0.60	34.69***

^***^ *p* < 0.001

### Mediation analysis

To estimate the indirect effect of self-concept clarity on working state through resilience as a mediator, a mediation study based on 5000 bootstrapping samples was carried out. Table 5 shows the results of the analysis of the mediating effect of resilience on self-concept clarity and job burnout. Both the direct (95% CI: [–0.38, –0.30]) and indirect (95% CI: [–0.05, –0.02]) effects of self-concept clarity on exhaustion were significant. The direct effect (95% CI: [–0.37, –0.30]) and indirect effect (95% CI: [–0.04, –0.01]) of self-concept clarity on cynicism were also significant. Similarly, the effect of self-concept clarity on reduced professional efficacy, including direct (95% CI: [− 0.11, − 0.04]) and indirect (95% CI: [− 0.12, − 0.08]) effects, was also significant.

**Table 5:** Mediation effects of resilience between self-concept clarity and job burnout

Model	Effect Type	Effect	Bootstrap SE	Bootstrap 95%CI lower limit	Bootstrap 95%CI upper limit
Exhaustion	Total effect	−0.37	0.02	−0.41	−0.33
	Direct effect	− 0.34	0.02	− 0.38	− 0.30
	Indirect effect	− 0.03	0.01	− 0.05	− 0.02
Cynicism	Total effect	− 0.35	0.02	− 0.39	− 0.32
	Direct effect	− 0.33	0.02	− 0.37	− 0.30
	Indirect effect	− 0.02	0.01	− 0.04	− 0.01
Reduced	Total effect	− 0.18	0.02	− 0.22	− 0.13
professional	Direct effect	− 0.08	0.02	− 0.11	− 0.04
efficacy	Indirect effect	− 0.10	0.01	− 0.12	− 0.08

Table 6 shows the results of the mediation analysis of resilience on self-concept clarity and work engagement. The direct effect of self-concept clarity on work engagement was significant (95% CI: [0.07, 0.14]), as was the indirect effect of resilience (95% CI: [0.10, 0.16]). These results indicate that resilience partially mediated the relationship between self-concept clarity and job burnout as well as the relationship between self-concept clarity and work engagement, supporting Hypothesis 4.

**Table 6:** Mediation effects of resilience between self-concept clarity and work engagement

Effect Type	Effect	Bootstrap SE	Bootstrap 95%CI lower limit	Bootstrap 95%CI upper limit
Total effect	0.23	0.02	0.19	0.27
Direct effect	0.10	0.02	0.07	0.14
Indirect effect	0.13	0.01	0.10	0.16

## Discussion

### Self-concept clarity as a resource for firefighters

The findings of this study extend the Job Demand–Resource model by demonstrating that self-concept clarity may be applied as a resource to ensure firefighters’ working states. Firstly, the findings are consistent with Hypothesis 1. The study discovered a link between self-concept clarity and firefighters’ job burnout. Research has shown that individuals with low levels of self-concept clarity have a certain vulnerability in adapting to chronic stress [47], while the inability to recover well under stress makes firefighters more prone to exhaustion [8]. On the other hand, firefighters with a clearer understanding of their own identity are better able to find meaning in their work, which reduces exhaustion and cynicism [48, 49]. Additionally, previous studies have demonstrated that self-concept clarity is closely associated with less negative coping styles [50], while reducing negative coping styles can resist reductions in professional efficacy [51]. In the current study, the results of correlation analysis showed also that self-concept clarity had a similar strength of correlation with exhaustion (*r* =  − 0.37) and cynicism (*r* = -0.35), and the weakest correlation with reduced professional efficacy (*r* =  − 0.18).

Secondly, the study discovered a positive correlation between firefighters’ self-concept clarity and work engagement, supporting Hypothesis 2. This is due to the fact that firefighters with higher levels of self-concept clarity focus more on their own positive characteristics and have a more favorable experience at work [48]. Additionally, individuals with a clearer sense of their own concept of worth have more adequate cognitive resources and are more likely to be proactive in finding solutions [52]. Previous findings suggest that such active coping has been linked to increased work engagement [17]. To summarize, the effect of self-concept clarity on job burnout and work engagement is consistent with the effect of resources on working state in JD–R theory [23], and it is reasonable therefore to consider self-concept clarity as a resource component.

### Relationships between resilience, self-concept clarity, and the working state of firefighters

The results of this study show a positive correlation between self-concept clarity and resilience, supporting Hypothesis 3. Consistent with previous studies [33, 53], this investigation demonstrates that the positive effect of self-concept clarity on resilience also exists in firefighters. As a positive resource, a clearer self-concept enhances an individual’s ability to regulate emotionally and psychologically, thereby improving their ability to adapt under high pressure and strengthening their resilience [31, 40, 54].

Moreover, resilience was found to mediate the relationship between self-concept clarity among firefighters and their working state, which is consistent with Hypothesis 4. While self-concept clarity promotes the development of resilience, higher resilience then helps individuals adapt well to stressful work environments, reducing job burnout [55, 56], and improving work engagement [38, 57]. Based on the theoretical framework of JD–R theory, it has been demonstrated that resilience, as a resource, can have a significant impact on job burnout [37]. Also based on JD–R theory, Lee and Kim [58] showed that resilience, as a resource, can promote work engagement. The present study demonstrates further that both self-concept clarity and resilience are protective factors for the working state of firefighters. Self-concept clarity can reduce firefighter job burnout and improve firefighter work engagement by improving their resilience. The results of the mediation models also indicate that self-concept clarity had a more direct effect on exhaustion and cynicism. However, its effect on reduced professional efficacy is more indirect; viz., through the mediating effect of resilience.

### Implications

This study has the following important implications. Firstly, it confirms the possibility of self-concept clarity as a resource, which not only expands JD–R theory, but also provides a new perspective on job burnout and work engagement among firefighters. Secondly, the research reveals that the mediating effect of resilience is the pathway through which self-concept clarity has an effect on their working state. Self-concept clarity can not only directly affect their working state, but can also reduce job burnout and improve work engagement by promoting resilience. These results suggest practical guidance for protecting firefighters by providing strategies for alleviating job burnout, improving work engagement, and helping firefighters to work in a better psychological state. Specifically, managers should strengthen the cultivation of self-concept clarity and resilience among firefighters in order to better maintain their working state. Previous studies have found that mindfulness practice is an effective means of improving self-concept clarity and resilience [59, 60]. Therefore, approaches to improving the working state of firefighters and the management of their psychological resources should consider the incorporation of mindfulness practice and other methods into their training in order to improve their self-concept clarity and resilience.

### Limitations and future research directions

This study has some limitations. First, the study sample included only male firefighters, and the lack of gender diversity limits the generalization of the findings. Future research should pay more attention to female firefighters to expand the scope of the current findings. Second, this study used a questionnaire survey to gather the data, which raises the question of subjectivity in the results. Future studies could evaluate burnout and other states among firefighters through experimental methods in order to enhance the objectivity of the results. Third, this study extends the resource component of JD–R theory by examining the role of self-concept clarity and resilience. Future studies could explore further whether there is an interactive relationship between self-concept clarity and resilience and other factors such as job demands. Fourth, this is a cross-sectional study. Future studies could research the causal relationship between variables through longitudinal tracking and other research designs. Fifth, the study looked at a particular profession, and it remains to be seen whether the findings would apply to other occupations. Therefore, future studies could test the results of this study in other occupational groups.

## Conclusion

This study has expanded Job Demand–Resource theory by demonstrating that self-concept clarity and resilience can be used as resources to reduce job burnout and enhance work engagement among firefighters. Self-concept clarity was negatively correlated with job burnout and positively correlated with work engagement. The study also found a positive association between self-concept clarity and resilience. As a special resource, self-concept clarity can not only directly affect the working state of firefighters, but can also influence job burnout and work engagement through the mediating effect of resilience.

## Data Availability

The datasets used and/or analyzed during the current study are available from the corresponding author on reasonable request.
